# Rearing system type affects baseline and post-vaccination immune gene expression in Atlantic salmon gills during smoltification

**DOI:** 10.17221/93/2025-VETMED

**Published:** 2026-07-27

**Authors:** Dagmar Mudronova, Adele Lagesen, Emil Pilipcinec, Natalia Chomova, Marek Ratvaj

**Affiliations:** Department of Microbiology and Immunology, University of Veterinary Medicine and Pharmacy in Košice, Košice, Slovak Republic

**Keywords:** cytokine, vaccination, water system

## Abstract

Abstract: Atlantic salmon undergo critical development before sea transfer, and vaccination during this stage is routine to build protective immunity. This study compared immune responses to vaccination in smolt-stage salmon reared in flow-through (FT) versus recirculating (REC) aquaculture systems. Ten clinically healthy fish per facility were collected one week before and two weeks after intraperitoneal vaccination with commercial multivalent vaccines. Using qPCR, we compared immune gene expression in the fish gills. Before the vaccination, REC fish exhibited higher relative expression of genes coding cathelicidin-1 (*cath-1*), interleukin-1β (*il-1β*), and tumour necrosis factor-α (*tnf-α*) than FT fish. Fourteen days after vaccination, REC fish showed increased expression of gene coding defensin (*def*) and decreased expression of gene for transforming growth factor*β* (*tgf-β*), while in FT fish, vaccination was associated with increased relative gene expression of *tnf-α*, *tgf-β,* and *il-10*. Within REC, *cath-1* expression declined postvaccination, concurrent with an increase in *il-10*, suggesting a possible counter-regulatory response; in FT, the coordinated rise of pro-inflammatory (*tnf-α*) and anti-inflammatory (*tgf-β, il-10*) transcripts is consistent with an activation followed by regulatory signalling. These transcriptomic patterns imply that baseline immune gene expression and post-vaccination gill transcriptomic responses differ between rearing water systems used during smoltification in Atlantic salmon.

In 2020, global aquaculture production reached 87.5 million tonnes, with the majority meant for human consumption. The most extensively farmed fish was Atlantic salmon (*Salmo salar*) (FAO 2022). Before Atlantic salmon enter the on-growing phase at sea, they undergo essential freshwater development in land-based smoltification facilities, where they progress from eggs to fry, start feeding, and grow until smoltification. Smoltification (parr-smolt transformation) enables salmonids to adapt from freshwater to seawater through morphological, physiological, biochemical, and behavioural changes (Lucas et al. 2019). Smolt quality – defined by seawater performance – is vital, as inferior quality is linked to increased post-transfer mortality and stunted growth. In fact, 32% of deaths within 180 days post-transfer are associated with smolt quality (Sommerset et al. 2023). Smolt production takes place in either flow-through (FT) or recirculating aquaculture systems (REC), depending on the design of the water intake (Stickney and Gatlin 2022). Data on juvenile losses in these facilities is limited compared to sea farms. Notably, fish weighing 0–3 grams account for ~45% of deaths in smoltification systems, with no distinction made between flow-through and recirculation system technologies in official reporting (Sommerset et al. 2023). Vaccination is now routine in modern salmonid farming, and Norway is a leader in vaccine development. Vaccines significantly reduce disease losses such as furunculosis (*Aeromonas salmonicida*), lower treatment costs (Gudding and Van Muiswinkel 2013), and reduce the use of antibiotics (Manage 2018). In salmonids, vaccines are administered during the freshwater phase while fish are immunocompetent but before pathogen exposure, allowing protective immunity to develop prior to seawater transfer (Lillehaug 2014).

Gill tissue represents a primary mucosal interface continuously exposed to environmental antigens, making it a sensitive indicator of both systemic immune activation and local environmental immune modulation in salmon (Auclert et al. 2024). This work aims to compare the immune gene expression in Atlantic salmon smolt gills, identifying whether the baseline immune status and post-vaccination responses differ between flow-through and recirculating aquaculture systems operated by the same company using the same river water intake.

## MATERIAL AND METHODS

### Ethical statement

The present work has been done in the country of Norway; therefore, it was performed in compliance with the Norwegian national legislation, Regulation on the operation of aquaculture facilities (Akvakulturdriftsforskriften) (Lovdata 2008) and the Regulation on the use of animals in experiments (Lovdata 2015), ensuring that proper methods that protect the welfare of animals were followed. This work was exempted from application for approval from the Norwegian Food Safety Authority according to the Regulation on the use of animals in experiments §6 Approval of experiment, which states that the requirement for approval does not apply to experiments that only involve euthanasia of animals to use organs or tissues from them.

### Experimental animals and sample collection

Smolts were sampled from two smoltification facilities in Bindal, Norway, both of which draw water from the same river: Saglifossen, a flow-through facility (FT) established in 1985, and Svaberget, a recirculating facility (REC) completed in 2022. The water parameters or microbial load were not measured for this study; however, the Norwegian aquaculture legislation mandates that fish farms regularly monitor and maintain suitable water parameters and conduct routine veterinary assessments.

Sampling occurred one week before and two weeks after vaccination, with ten fish per facility and timepoint randomly selected, limited to individuals of suitable size and clinically healthy appearance.

The fish were vaccinated with ALPHA JECT Micro 1 PD, ALPHA JECT Micro 6, Alpha ERM Salar, and ALPHA JECT Moritella (PHARMAQ, Overhalla, Norway). All the vaccines were administered via intraperitoneal injection according to the manufacturer’s instructions. ALPHA JECT Micro 1 PD for immunisation against pancreas disease (PD). ALPHA JECT Micro 6 against *Aeromonas salmonicida* (furunculosis), *Vibrio salmonicida* (coldwater vibriosis), *Listonella anguillarum* serotype O1 and O2a (vibriosis), *Moritella viscosa* (winter sores), and infectious pancreatic necrosis (IPN). Alpha ERM Salar against *Yersinia ruckeri,* serotype O1b (enteric red mouth disease). ALPHA JECT Moritella against *Moritella viscosa* (winter sores).

The fish were first subjected to immersion in a benzocaine anaesthetic, Benzoak vet. (ACD Pharmaceuticals, Leknes, Norway), dissolved in a bucket of water at a dose of 30–40** **mg/l of water. The fish were deemed properly anaesthetised when all activity and reactivity were absent, equilibrium had been lost, the gill ventilation rate was rare, and no eye movements were detected (Sneddon 2012). They were then euthanised by exsanguination, by cutting the ventral aorta. All fish were examined for external deformities before being opened lateroventrally to see if there were any gross lesions on the internal organs, as well as noting the presence of feed in the digestive tract, to ensure only seemingly healthy fish were sampled. A piece of tissue approximately 4 mm from the second gill arch was then dissected out and placed in Eppendorf tubes with 1 ml of RNAlater (Thermo Fisher Scientific, Waltham, MA, USA), then stored at –18 **°**C until further processing.

### Gene expression study

Homogenisation of the gill tissue was done by bead milling. Glass beads with a diameter of 1.4 mm were added to tubes, together with a lysis buffer (700 μl TRK Lysis Buffer from E.Z.N.A.^®^ Total RNA kit I) and no more than 30 mg of tissue. A Precellys 24 Tissue Homogeniser (Bertin Technologies, Montigny-le-Bretonneux, France) was then used at 6 000 rpm in 2 × 30 s cycles with a 15 s break between.

The RNA was isolated using E.Z.N.A.^®^ Total RNA kit I (Omega Bio-tek, Norcross, GA, USA) following the manufacturer’s instructions. In brief, after homogenisation, a clear supernatant was mixed with one volume of 70% ethanol, transferred to a HiBind^®^ RNA Mini Column, and centrifuged at 10 000 *g* for 1 minute. The filtrate was discarded, and the samples were then washed using 500 μl RNA Wash Buffer I and centrifuged at 10 000 *g* for 30 seconds. Then two consecutive rounds of washing with 500 μl RNA Wash Buffer II and centrifuged at 10 000 *g* for 1 minute. Columns were dried by centrifugation at maximum speed for 2 minutes. The isolated RNA was eluted from the membrane using 40 μl of nuclease-free water. Purity and concentration were measured using a spectrophotometer, Nanodrop 8000 (Thermo Scientific, Waltham, MA, USA) at 260/280 nm, and integrity was evaluated using gel electrophoresis. Transcription of 1 000 ng of RNA to cDNA was done by a QuantiTect^®^ Reverse Transcription Kit (QIAGEN, Hilden, Germany), used according to the manufacturer’s manual. A step of gDNA removal is part of the protocol. cDNA was stored at –18 °C before qPCR.

PCR reactions (10 μl total volume) contained 5 μl iQ^TM^ SYBR Green I Supermix (BioRad, Hercules, CA, USA), 0.5 μl each of forward and reverse primers (Table 1), and 4 μl cDNA with a concentration of 10 ng/μl. Amplification was performed on a CFX96 Touch thermocycler (BioRad, Hercules, CA, USA) in duplicate, with no template controls. Cycling conditions were: 95 °C for 3 min, followed by 40 cycles of 95 °C for 15 s and 60 °C for 30 seconds. A melting curve analysis verified amplification of a single specific product. Relative gene expression was measured as ΔΔCt (±SD) using the Pfaffl Method in Microsoft Excel (Microsoft, Redmond, WA, USA) with *ubiquitin* serving as a reference gene.

**Table 1:** Sequences of primers used in this study

Gene	Sequence	Reference
Interleukin-1β (*il-1β*)	F: GCTGGAGAGTGCTGTGGAAGA	Hynes et al. (2011)
	R: TGCTTCCCTCCTGCTCGTAG	
		
Interleukin-10 (*il-10*)	F: CGCTATGGACAGCATCCT	Mutoloki et al. (2010)
	R: AAGTGGTTGTTCTGCGTT	
		
Transforming growth factor-β (*tgf-β*)	F: GGTACTGGCTCTGTATAAGCAC	Kiron et al. (2016)
	R: CTGCTCCACCTTGTGTTGTC	
		
Cathelicidin-1 (*cath-1*)	F: CTAGCAACAACCTGAACACTG	Kiron et al. (2016)
	R: CTTCTTGTCCGAATCTTCTGCATA	
		
Tumour necrosis factor-α (*tnf-α*)	F: TGCTGGCAATGCAAAAGTAG	Vasanth et al. (2015)
	R: AGCCTGGCTGTAAACGAAGA	
		
Defensin (*def*)	F: CAGTGCAAGCTGATGACACA	Sorensen et al. (2021)
	R: CCTGGGCATAGCAGTACTTG	
		
Ubiquitin (*ubi*)	F: AGCTGGCCCAGAAGTACAACTGTG	Kiron et al. (2016)
	R: CCACAAAAAGCACCAAGCCAAC	

### Statistical analysis

The statistical analysis used in this work was ANOVA with Tukey post hoc test in JASP software v0.95.3. (JASP Team, Amsterdam, The Netherlands).

## RESULTS AND DISCUSSION

Using qPCR, the expression of genes encoding molecules involved in the immune response was analysed in the gills of salmon reared in different water systems before and after vaccination. We focused on the gills of fish as it is one of the first points of contact of the fish organism with antigens from the environment and is also a sensitive indicator of systemic response. Antimicrobial peptides, including cathelicidin-1 and defensin were included as markers of mucosal innate immune activation, given their constitutive expression in gill tissue and documented responsiveness to immune stimulation (Bridle et al. 2011; Harte et al. 2020). Pro-inflammatory cytokines (IL-1β, TNF-α) and regulatory anti-inflammatory cytokines (TGF-β, IL-10) were selected to characterise the balance between immune activation and regulatory signalling.

**Figure 1:**
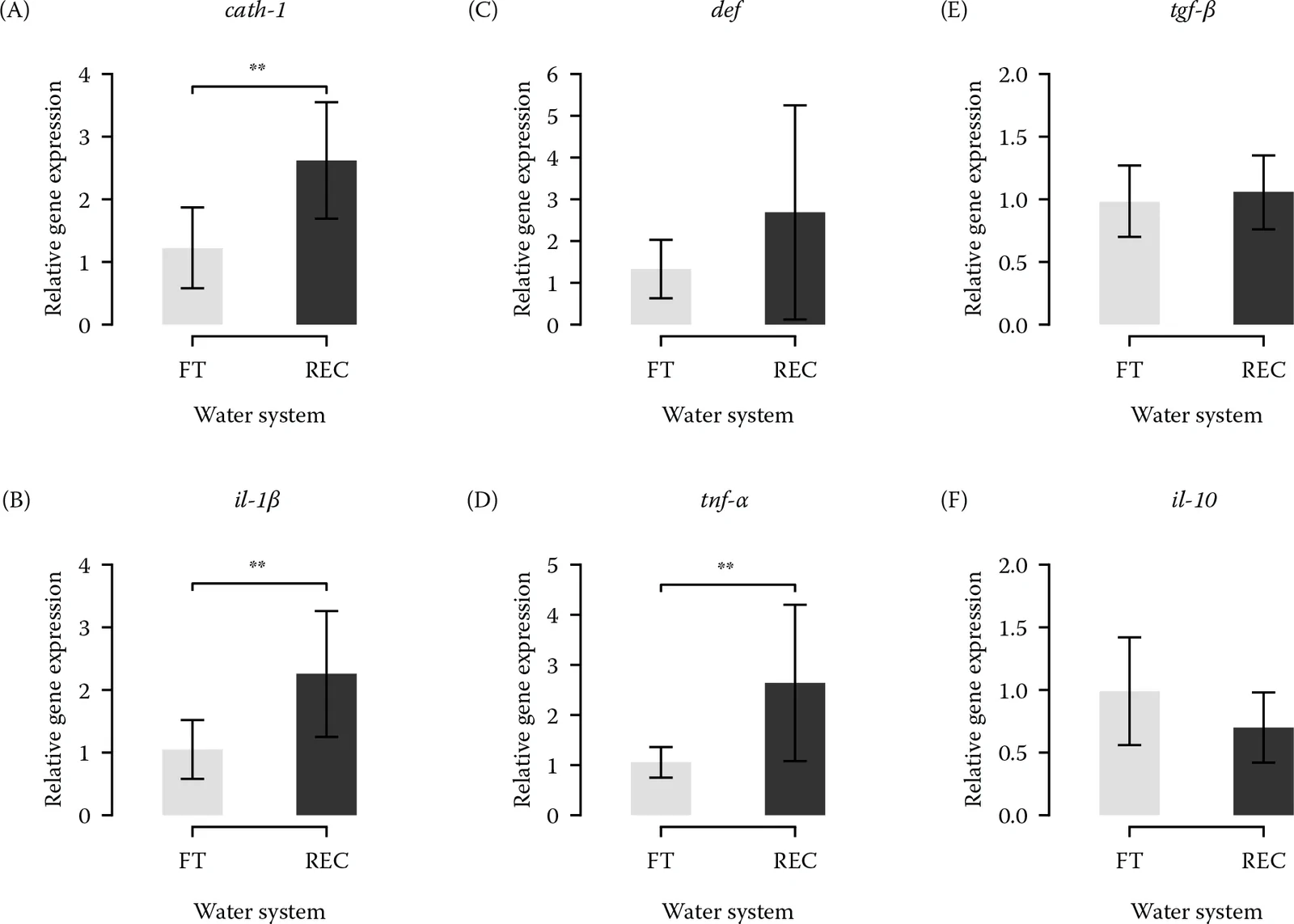
Relative expression of genes in salmon before vaccination (A) *cath-1*, (B) *il-1β*, (C) *def*, (D) *tnf-α*, (E) *tgf-β*, and (F) *il-10* in the gills of Atlantic salmon smolts reared in flow through (FT) and recirculating (REC) aquaculture systems before the vaccination (*n* = 10 per group) Results presented as relative gene expression ± SD; ** *P* ≤ 0.01

Before the vaccination, a significantly higher relative gene expression of *cath-1*, *il-1β*, and *tnf-α* was observed in the REC system compared to the FT system (Figure 1A,B,D). IL1 and TNF are pro-inflammatory cytokines in fish linked to inflammatory processes (Wangkahart et al. 2022), and they are often expressed together (Zou and Secombes 2016). Their elevation in the REC group suggests a state of basal mucosal immune activation before vaccination. This activation is likely driven by the inherent mechanics of recirculating systems; despite filtration, REC environments typically maintain a higher environmental antigenic load (e.g., microbial density or organic particulates) than flow-through systems (Auclert et al. 2024). However, further testing of water chemistry and microbiology would be necessary to confirm this hypothesis. Cathelicidin-1 plays an immunomodulatory role, facilitating cytokine release and exhibiting antimicrobial activity (Bridle et al. 2011). Cathelicidins are known to have a broad range of antimicrobial effects, being active against bacteria (both Gram-negative and Gram-positive), viruses, parasites, and fungi. One study found that healthy tissues of Atlantic salmon constitutively express *cath-2*, whereas *cath-1* is only expressed following bacterial infection (Bridle et al. 2011). The observed increase in *cath-1* gene expression in REC fish is consistent with greater mucosal immune stimulation, though the specific environmental drivers cannot be identified without water quality data. Along with the co-expression of the pro-inflammatory cytokines genes, these results show that prior to vaccination, gill immune gene expression differed between the two rearing systems, with REC fish showing a pattern consistent with greater mucosal immune activation.

**Figure 2:**
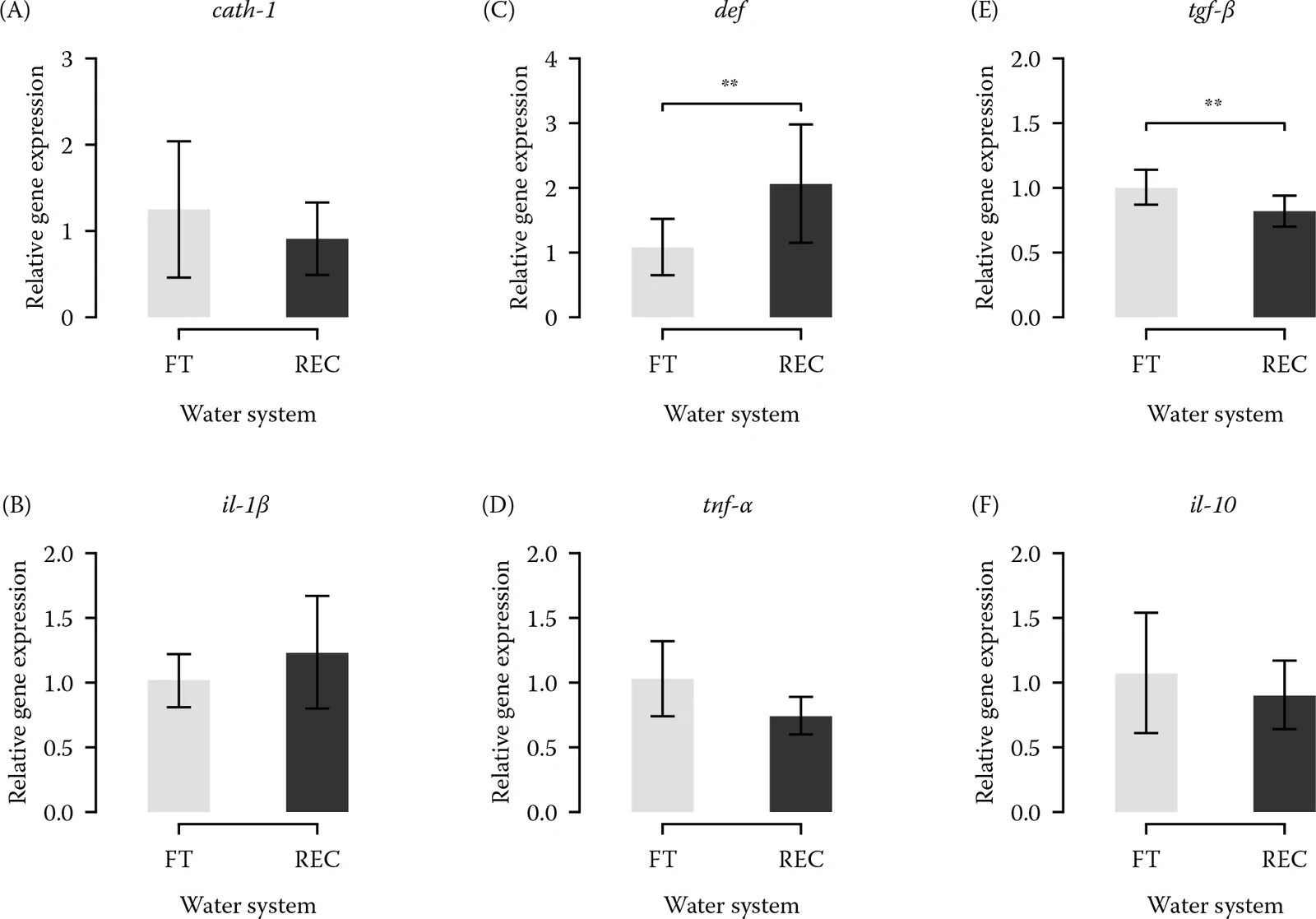
Relative expression of genes in salmon after vaccination (A) *cath-1*, (B) *il-1β*, (C) *def*, (D) *tnf-α*, (E) *tgf-β*, and (F) *il-10* in the gills of Atlantic salmon smolts reared in flow through (FT) and recirculating (REC) aquaculture systems after the vaccination (*n* = 10 per group) Results presented as relative gene expression ± SD; **P ≤ 0.01

After the vaccination, we observed a significantly increased relative gene expression in the REC group in the case of *def* (Figure 2C) and a significant decrease in expression of *tgf-β* (Figure 2E) compared to the FT group. Defensin is an antimicrobial peptide that has an important role in the mucosal innate immune system of Atlantic salmon (Harte et al. 2020). Their presence in the secreted mucus is important for its antimicrobial effects, and they also possess immunomodulatory functions (Shabir et al. 2022). Overall increase in the expression of this gene could benefit the organism in providing a better defence, if this change translates to the antimicrobial peptide synthesis. The difference observed between water systems post vaccination can result from different levels of immune stimulation from the environment in the REC compared to the FT system as was discussed in the results before the vaccination. TGF-β has an immunomodulatory function with both suppressive and stimulating effects. TGF-β can be synthesised by all leukocytes and has different and vast effects on immune cells, such as the inhibition of NK-cells, macrophages, and neutrophils, as well as the apoptosis of different T-cells. TGF-β was found to have significant suppressive effects on T-cell proliferation as well as decreasing pro-inflammatory cytokine expression. All these effects of increased TGF-β expression may render the fish more susceptible to bacterial infections, but they are also important in preventing excessive inflammatory responses and autoimmune diseases (Zhang et al. 2023). Since the vaccination itself activates the immune system, an expected response would be the increase in the expression of *tgf-β* to limit activation in order to prevent damage to tissues. Compared to the fish in the FT group, the REC fish may therefore have longer sustained pro-inflammatory reaction to vaccines. This increase in *tgf-β* was not associated with the elevated expression of any other inflammatory cytokines measured during this sampling, suggesting that this may be a slower return to basal levels.

**Figure 3:**
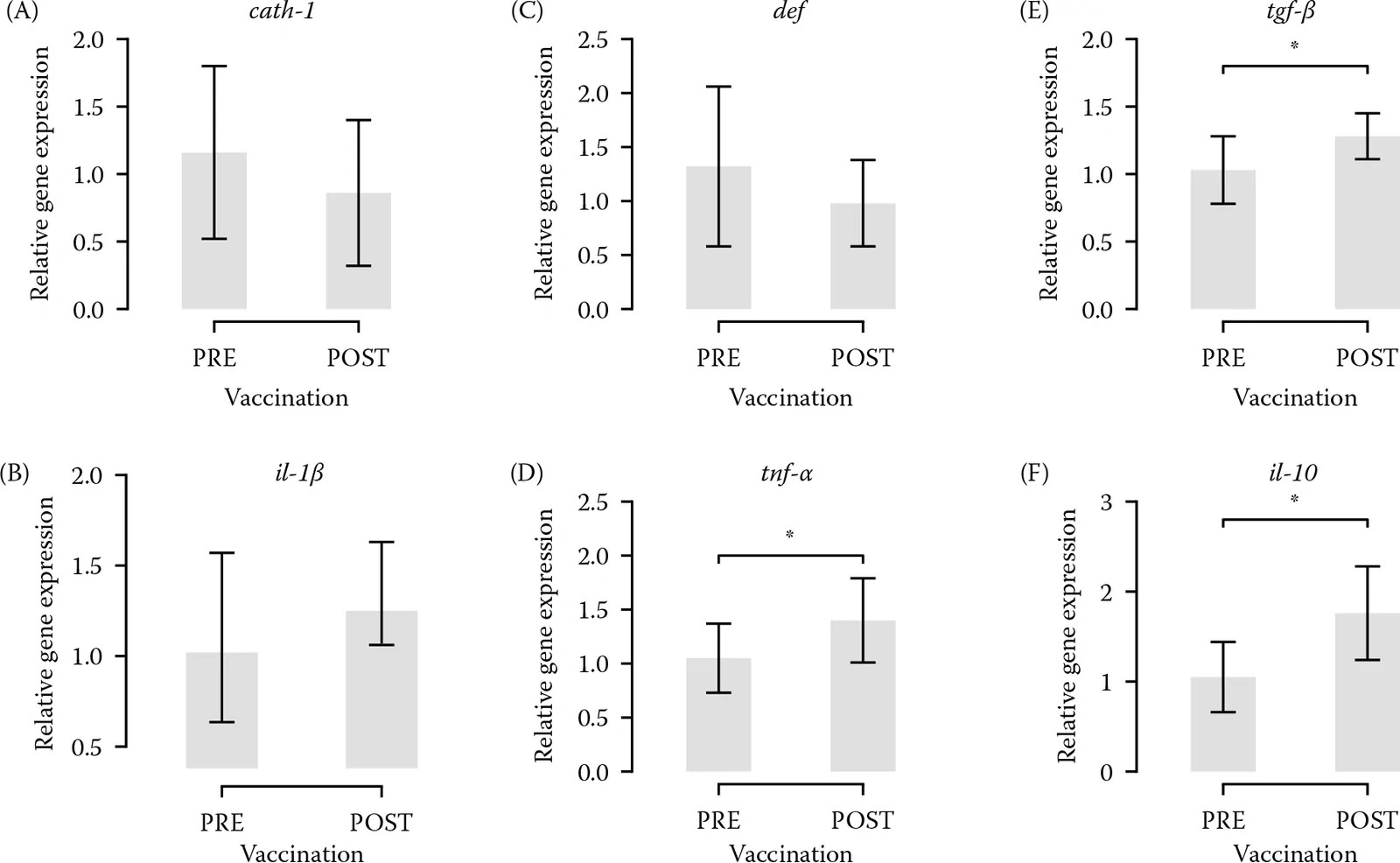
Relative expression of genes in salmon in a flow through system (A) *cath-1*, (B) *il-1β*, (C) *def*, (D) *tnf-α*, (E) *tgf-β*, and (F) *il-10* in the gills of Atlantic salmon smolts reared in flow through (FT) aquaculture systems before the vaccination (PRE) and after the vaccination (POST) (*n* = 10 per group) Results presented as relative gene expression ± SD; *P ≤ 0.05

In FT fish, vaccination was associated with concurrent upregulation of *tnf-α* alongside *tgf-β* and *il-10* gene expression (Figure 3D,E,F). This pattern is consistent with a classical two-phase immune response – an initial pro-inflammatory activation represented by *tnf-α* upregulation, followed by regulatory counter-signalling through *tgf-β* and *il-10* to restore immune homeostasis and prevent excessive inflammation (Saraiva et al. 2020; Zhang et al. 2023). The coordinated nature of this response suggests that FT fish, starting from a lower baseline immune activation, mounted a structured and balanced immune response following vaccination. The absence of significant changes in *cath-1*, *il-1β*, and *def* in FT fish post-vaccination may reflect the immune state of mucosal innate defences, which were not strongly recruited by intraperitoneal antigen delivery. Samples were collected at 14 days post-vaccination, and it should be noted that gene induction is time-dependent; earlier or later sampling may have revealed additional expression changes not captured at this time point.

**Figure 4:**
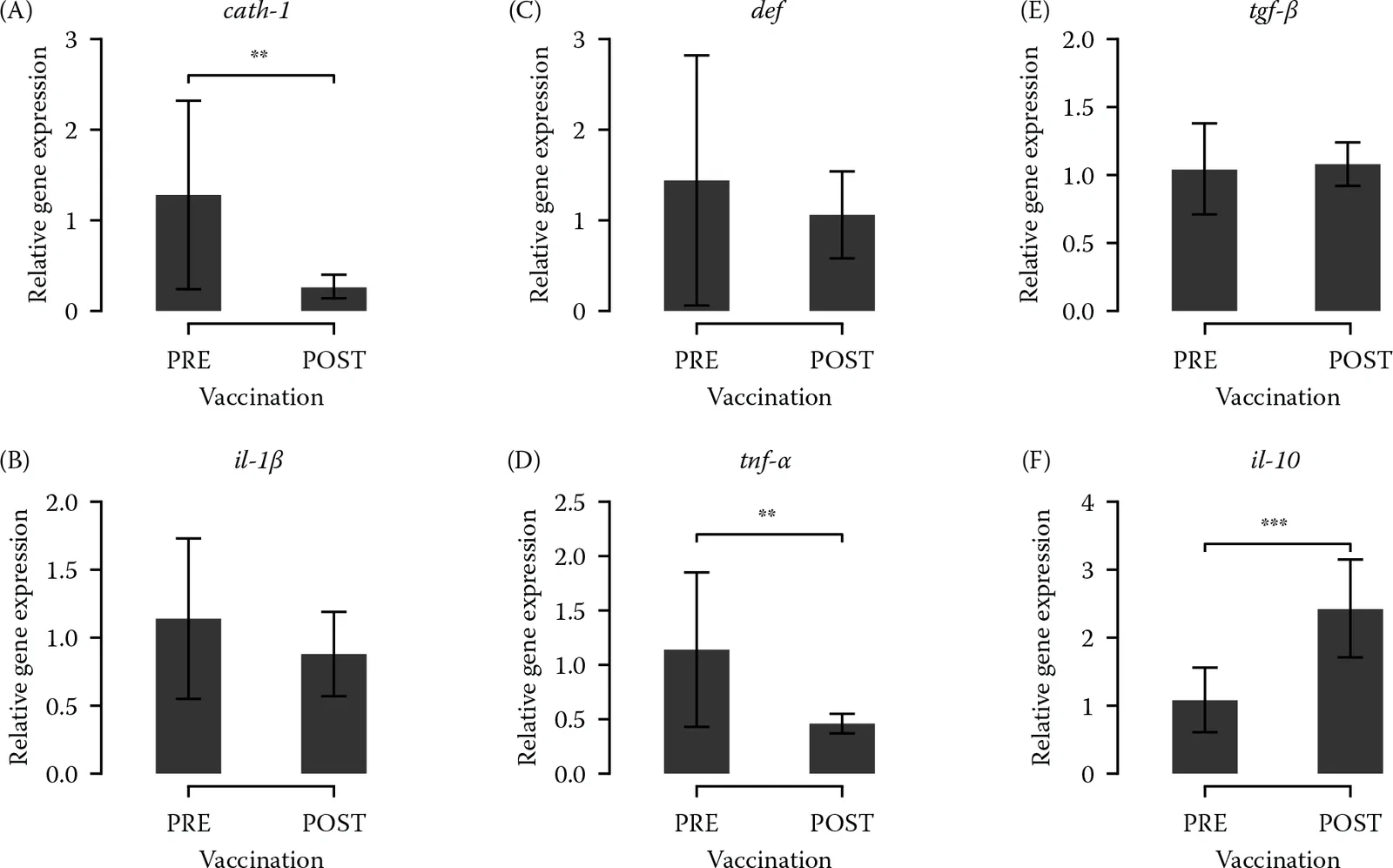
Relative expression of genes in salmon in a recirculating system (A) *cath-1*, (B) *il-1β*, (C) *def*, (D) *tnf-α*, (E) *tgf-β*, and (F) *il-10* in the gills of Atlantic salmon smolts reared in recirculating (REC) aquaculture systems before the vaccination (PRE) and after the vaccination (POST) (*n* = 10 per group) Results presented as relative gene expression ± SD; **P ≤ 0.01; ***P ≤ 0.001

In REC fish, the post-vaccination period was characterised by a significant decrease in *cath-1* expression concurrent with upregulation of *il-10* (Figure 4A,F), alongside a downregulation of *tnf-α* (Figure 4D). Rather than reflecting a direct vaccine effect, this pattern should be interpreted in the context of the elevated baseline immune activation observed in REC fish prior to vaccination (Figure 1A,B,D). The post-vaccination decrease in* cath-1* and suppression of *tnf-α* may therefore represent active restoration of immune homeostasis from an elevated baseline, rather than vaccine-specific immune suppression. The upregulation of *il-10*, a regulatory cytokine with inhibitory effects on pro-inflammatory signalling including *il-1β* and *tnf-α* (Alvarez et al. 2016; Huo et al. 2019), supports this interpretation – a high *il-10* expression may counteract elevated innate immune responses, contributing to the normalisation of *cath-1* and *tnf-α* observed at 14** **days post-vaccination. Together, these findings suggest that in REC fish, the post-vaccination transcriptomic response was dominated by regulatory rather than pro-inflammatory signalling, a pattern that may reflect the elevated pre-vaccination immune gene expression observed in this group rather than a direct response to the vaccine antigens.

Our results show that fish in the recirculating aquaculture system (REC) exist in a state of heightened basal immune alertness prior to vaccination, characterised by the induction of non-constitutive antimicrobial peptide (*cath-1*) and pro-inflammatory cytokines (*il-1β, tnf-α*) relative to flow-through (FT) fish. Following vaccination, the two systems exhibited different molecular patterns of gene expression. The FT fish mounted a structured, two-phase activation and resolution response (upregulation of *tnf-α* alongside *tgf-β* and *il-10*). The REC fish showed a sharp downregulation of *cath-1* and *tnf-α* post-vaccination, driven by *il-10* mediated suppression of their elevated baseline.

The distinct immunological responses suggest that the aquatic environment during smoltification shapes the immunological context in which vaccination occurs, independently of the vaccine itself. While the present study was not intended to determine which rearing system produces a superior immune response to vaccination, the characterisation of system-dependent immune gene expression profiles established here provides a necessary foundation for future studies incorporating serological, survival, and multi-timepoint endpoints under controlled experimental conditions. Further testing would be necessary to confirm whether these changes are significant for the developing fish with the potential to affect the health of salmon in the future, when they are being reared in the sea, and to confirm whether the flow-through system is preferred for rearing the fish during smoltification.
